# The impact of COVID-19 on the everyday life of blind and sighted individuals

**DOI:** 10.3389/fpsyg.2022.897098

**Published:** 2022-10-28

**Authors:** Monica Gori, Giorgia Bertonati, Emanuela Mazzoni, Elisa Freddi, Maria Bianca Amadeo

**Affiliations:** ^1^Unit for Visually Impaired People, Italian Institute of Technology, Genova, Italy; ^2^DIBRIS, Università degli studi di Genova, Genova, Italy; ^3^PREPOS Studio Associato, Lucca, Italy

**Keywords:** blindness, COVID-19, daily habits, personal experience, social life

## Abstract

The COVID-19 pandemic caused unexpected and unavoidable changes in daily life worldwide. Governments and communities found ways to mitigate the impact of these changes, but many solutions were inaccessible to people with visual impairments. This work aimed to investigate how blind individuals subjectively experienced the restrictions and isolation caused by the COVID-19 pandemic. To this end, a group of twenty-seven blind and seventeen sighted people took part in a survey addressing how COVID-19 impacted life practically and psychologically, how it affected their daily habits, and how it changed their experiences of themselves and others. Results demonstrated that both sighted and blind individuals had a hard time adapting to the new situation. However, while sighted people struggled more with personal and social aspects, the frustration of the blind population derived mostly from more practical and logistical issues. Likely as consequences, results showed that blind people engaged more in their inner life and experienced fear and anger as main emotions. This study suggests that changes in life associated with COVID-19 have been subjectively experienced differently based on the presence or not of blindness, and that tailored future interventions should be considered to take care of the different needs of blind individuals.

## Visual impairment and quality of life

There are about 300 million people with visual impairment in the world. The International Classification of Diseases 10 (ICD-10) classifies blindness as a condition of visual impairment with a distance visual acuity worse than 1/20. Although it has been demonstrated that people with visual disabilities can cope with the lack of vision in several domains of the everyday life, they still face some difficulties in some aspects of life. First, blindness is associated with higher risk for encountering other disabilities (Fenwick et al., 2012; Cumberland et al., 2016) and health-related issues (Crews et al., 2016; Cumberland et al., 2016). In addition, blind individuals often experience work and financial difficulties (Fenwick et al., 2012; Gordois et al., 2012; Cumberland et al., 2016), as well as negative interpersonal events (Brunes et al., 2018, 2019a; Brunes and Heir, 2021). These factors, in some cases, may cause psychological problems in the blind population (Cimarolli et al., 2016; Zheng et al., 2017; Brunes and Heir, 2020). In this regard, adults with visual impairments tend to feel lonelier and less supported than sighted people (Brunes et al., 2018, 2019b; Jackson et al., 2019). Given that social learning is often mediated by visual feedback (such as through facial expressions, gestures, and eye contact), a lack of vision may limit some social competence of blind individuals, who may appear less willing to start social interactions with others, may feel more isolated, and may need different social requirements than sighted people (Sharon et al., 2000; Krishna et al., 2008). These social difficulties are associated with a higher risk of developing symptoms of anxiety and depression (Evans et al., 2007; Schilling et al., 2013; Frank et al., 2019; Morse, 2019; Yu and Liljas, 2019). These problems are even more likely to occur if the visual impairment is acquired later in life (e.g., Horowitz, 2004; Zheng et al., 2017). All together, these factors unavoidably have an influence on the quality of life of people with visual impairment, which is generally lower than that of sighted individuals (Vu et al., 2005; Khorrami-Nejad et al., 2016; Tseng et al., 2018; Xu et al., 2018; Jones et al., 2019).

### COVID-19 pandemic

The COVID-19 pandemic caused unexpected and unavoidable changes in our lives. This was especially true in Italy. During the first wave of the COVID-19 crisis, people remained isolated for three months in their house, with limited possibilities to go out or to see other people. This made all everyday activities more complex. Government, communities, and businesses developed solutions, such as online apps for food shopping or remote working procedures. However, most of these solutions were inaccessible to visually impaired people. Indeed, people with disabilities have been among the most severely affected by lockdowns (Jalali et al., 2020; Lund et al., 2020; Mbazzi et al., 2020; Safta-Zecheria, 2020). Social distancing and other limitations of the so-called ‘new normal’ were particularly difficult for those with a visual impairment. People with blindness generally cope with their lack of sight by relying on their other senses, which became more difficult under COVID-19 health measures such as physical distancing, wearing facemasks, and using gloves (Giudice, 2020; Rizzo et al., 2021; Suraweera et al., 2021). A recent study reported that the lockdown hindered blind people in accessing daily support needs, remote work for higher education or careers, shopping, and leisure (Gombas and Csakvari, 2021). According to a survey conducted by the Royal National Institute of Blind People in May 2020, 66% of blind and partially sighted people felt less independent after the lockdown; 80% reported the way they shopped for their essential items changed, with most respondents concerned about accessing food; and 26% of respondents declared they had struggled to obtain written information in a format that they could read.^1^ Finally, a recent study showed that visually impaired people perceived increased barriers to physical exercise during the pandemic emergency, leading to a reduced ability to exercise by visually impaired individuals, especially during the initial stages of the lockdown (Richardson et al., 2022).

Although there is no doubt that the COVID-19 emergency had economic, social, and psychological consequences on the blind population (Suraweera et al., 2021), it is important to understand how the COVID-19 crisis subjectively affected visually impaired individuals’ feelings, their experiences of themselves and others, and their everyday habits.

### The goal of the study

In this work, we investigated the effect of the lockdown and social distancing on different dimensions of life right after the first months of COVID-19 in Italy, June 2020. Twenty-seven blind and seventeen age-matched sighted individuals were involved in a telephone survey. Given that blind people already report a feeling of social isolation and a lack of social support (Sharon et al., 2000; Krishna et al., 2008), a preliminary open-ended question was posed to address general psychological and practical issues experienced during COVID-19. A subsequent questionnaire on daily routine, social life, and sleep habits was proposed since blind people sometimes report some difficulties in carrying out some everyday activities (Pardhan et al., 2015) and may be at risk of poor sleep quality and quantity (Peltzer and Phaswana-Mafuya, 2017; Hartley et al., 2018). A final questionnaire was administered to assess how COVID-19 impacted subjective self-experience and the experience of others in both sighted and blind participants.

Results suggest that the change of habits and isolation in the lockdown period under COVID-19 produced some different effects in sighted and blind individuals, evidencing the need for tailored interventions to support blind individuals in everyday activities.

## Materials and methods

### Participants

Seventeen sighted and twenty-seven blind adults took part in the study (mean age ± standard deviation: 40.82 ± 16.13 years old for sighted individuals, 46.92 ± 13.99 years old for blind individuals; female: 12 for sighted individuals, 10 for blind individuals). Seventeen sighted individuals completed the survey. Twenty-seven blind participants joined the open-ended question stage and Questionnaire 2, while twenty-six blind individuals completed Questionnaires 1 (one blind participant dropped out). Clinical details of blind people are reported in Table 1. None reported impaired hearing abilities or neurological, cognitive, or sensory-motor deficits except for total blindness. The research protocol was approved by the local health service ethics committee (Comitato Etico, ASL3 Genovese, Italy) and conducted according to the Declaration of Helsinki. With the approval of the ethics committee, participants had the possibility to sign the informed consent declaration online.

**Table 1:** Clinical details of blind participants.

Blind participant	Gender	Age	Age of blindness onset	Aetiology	Residual vision
1	M	29	19 years old	Leber Amaurosis	Light perception
2	F	30	6 years old	Optic Nerve Tumour	Light perception
3	M	32	17 years old	Corneal Opacity	No vision
4	F	33	birth	Retinopathy Of Prematurity	No vision
5	M	60	18 years old	Marfan Syndrome	Light perception
6	M	28	birth	Leber Amaurosis	No vision
7	F	60	40 years old	Retinitis Pigmentosa	Light perception
8	M	32	20 years old	Degenerative Oculopathy	Light perception
9	F	31	birth	Retinopathy Of Prematurity	No vision
10	F	55	46 years old	Leber Amaurosis	No vision
11	F	43	birth	Microphthalmia With Congenital Cataract	No vision
12	M	48	birth	Atrophy Optic Nerve	No vision
13	M	55	40 years old	Retinitis Pigmentosa	Light perception
14	F	53	40 years old	Retinitis Pigmentosa	Light perception
15	M	55	birth	Retinopathy Of Prematurity	No vision
16	M	57	14 years old	Optic Chiasm Tumour	No vision
17	M	60	11 years old	Uveitis	Light perception
18	F	30	birth	Microphthalmia	No vision
19	M	72	14 years old	Congenital Glaucoma	No vision
20	F	31	birth	Retinitis Pigmentosa	Light perception
21	M	61	birth	Congenital Glaucoma	No vision
22	M	62	20 years old	Congenital Glaucoma	No vision
23	M	70	51 years old	Retinal Detachment	No vision
24	M	44	birth	Retinopathy Of Prematurity	No vision
25	F	41	30 years old	Retinitis Pigmentosa And Stalgat Syndrome	Light perception
26	M	48	6 years old	Congenital Glaucoma	No vision
27	M	68	39 years old	Retinitis Pigmentosa	Light perception

M = male, F = female.

### Procedure

At the end of the first lockdown due to COVID-19 restrictions in Italy, where closure ran from March to May 2020 with data collected in June and July 2020, blind and sighted people subscribed to our laboratory mailing list were invited to participate in this study. They were informed about the specific objectives of the survey during a phone call. Those who accepted received the informed consent declaration form, fixed a phone appointment with one researcher, and were called on the appointed day and time. During the call, all participants answered an open-ended question and two questionnaires orally together with the researcher. To avoid possible confounds, one researcher performed all the phone calls. Respondents were allowed to take their time in answering the interview questions, thus the phone call varied from 20 to 50 min depending on the participant. Phone call was chosen as the method by which to collect data to avoid individual difficulties related to technological skills, to encourage participation, and to minimize the risk of dropping before the end of the survey. Quantitative data were subsequently analysed with RStudio (RStudio Team, 2015).

### Preliminary open-ended question

Participants were asked to respond to the following open question: “What was the greatest difficulty you encountered in your daily life during the COVID-19 lockdown, both on a practical and psychological level?.” No time limit was given. This question was intended to give the opportunity to provide a personal point of view on individual difficulties in the critical situation associated with the COVID-19.

### Questionnaire 1

To further investigate how COVID-19 impacts people’s lives, we asked additional questions about individuals’ daily routines, social life, and sleep habits. Table 2 shows the list of questions.

**Table 2:** Questionnaire 1 questions list: items were divided into three main categories: daily routine, social life, and sleep habits.

Questions	Type of response
a) Daily routine
*“How much do you think your daily routine changed with COVID-19?”*	10-point Likert scale
*“How many times on average per week have you left your house since the COVID-19 lockdown?”*	Number
*“How many times on average per week did you go out from your house before COVID-19?*	Number
b) Social life
*“Has the number of people you are virtually in touch with (calls, chats, social networks,* etc.*) changed since the COVID-19 lockdown?”*	5-point Likert scale
*“Has the number of people you have in-person social interactions changed since the COVID-19 lockdown?”*	5-point Likert scale
c) Sleep habits
*“Do you think the quality of your sleep changed since the COVID-19 lockdown?”*	10-point Likert scale
*“Do you think the amount of your sleep changed since the COVID-19 lockdown?”*	10-point Likert scale
*“How many hours do you sleep on average per night since the COVID-19 lockdown?”*	Number
*“How many hours did you sleep on average per night before COVID-19?”*	Number
*“Do you need a lot of time to fall asleep since the COVID-19 lockdown?”*	Yes/No
*“Did you need a lot of time to fall asleep before COVID-19?”*	Yes/No
*“Do you often wake up during the night since the COVID-19 lockdown?”*	Yes/No
*“Did you often wake up during the night before COVID-19?”*	Yes/No
*“Do you often wake up early, before the alarm, since the COVID-19 lockdown?*	Yes/No
*“Did you often wake up early, before the alarm, before COVID-19?”*	Yes/No

For some questions, participants had to indicate on a ten-point Likert scale how much a particular aspect of their life changed due to COVID-19, with one showing no changes at all, five and six representing moderate changes, and 10 indicating drastic changes. In addition, two questions on social life required participants to answer on a 5-point Likert scale, as we evaluated whether the number of people participants interacted with had reduced or increased during the COVID-19 pandemic through explicit statements from which participants could select (e.g., 1-the number of people you were virtually in touch was drastically reduced, 2-the number of people you were virtually in touch was slightly reduced, 3-the number of people you were virtually in touch did not change, 4-the number of people you were virtually in touch was slightly increased, 5-the number of people you were virtually in touch was drastically increased). Some other questions required participants to report an actual number to estimate how many times/h they faced a specific event in the current moment and before COVID-19. Lastly, some questions required Yes/No answers.

### Questionnaire 2

The primary goal of this questionnaire was to understand whether the restrictions of COVID-19 affected blind people’s personal experiences of themselves and others differently, compared with sighted people. We designed this questionnaire inspired by a psychological test developed by PREPOS (Prevenire è Possibile), an Italian treatment group led by Vincenzo Masini initially born for drug addiction (Masini, 2009). Research by the PREPOS group aimed to understand the choices addicts would make in their specific kind of drug and the ultimate roots of their addiction (Masini, 2005a; Masini et al., 2017). Starting from the six emotions identified by Ekman (Ekman, 1999), Masini added another emotional state, quietude, and proposed an educational path where emotions are considered as feelings and each feeling, intensified, resulted in a personality category (Masini, 2005b). According to the model developed by PREPOS, emotional experiences in formative early-year relationships influence the ‘personality profile’ of individuals, or their predominant way of feeling during adulthood. Difficulties in emotional experiences drive individuals to avoid certain ways of feeling and increase predisposition to others.

Thus, the model divides personalities into seven categories according to the core emotion (s) the individual experienced during their formative years:

- Stingy: fear as the core emotion experienced. Fear is the predominant feeling that drives individuals to be anxious and ready to defend themselves from danger. It is a personality based on control and responsibility.
- Ruminant: anger as the core emotion experienced. Anger is the predominant feeling that drives individuals to be responsive and resentful. It is a personality based on action and commitment.
- Delusional: detachment and surprise as core emotions experienced. The awareness of the distance between oneself and others generates a feeling of surprise that drives to distance oneself from the external world. It is a personality based on mentalization and freedom.
- Partier: pleasure as the core emotion experienced. The pursuit of pleasure is the predominant feeling that drives individuals to lose the boundaries of the self and melt with others in the search of adventure, novelty and satisfaction. It is a personality based on involvement and generosity.
- Apathetic: quietude as the core emotion experienced. The lack of emotions is the predominant feeling that drives individuals to avoid any external stimulus. It is a personality based on shutdown and, consequently, peace.
- Invisible: embarrassment as the core emotion experienced. The predominant feeling of embarrassment drives people to withdraw and control internal emotional states. It is a personality based on sensitivity and resilience.
- Adhesive: attachment as the core emotion experienced. The need of emotional closeness is the predominant feeling that drive individuals to look for the presence of others. It is a personality based on interaction and loyalty.

From these seven personality categories, the founder created a specific questionnaire able to investigate personality and interpersonal relationships across groups of people. The questionnaire has been applied in Italy in different contexts, such as schools, working environments, sociology, psychology, psychiatry, medicine, and counselling. It investigates three important aspects of adult life: the self (i.e., the relationships with one-self), the world (i.e., the relationships with the external environment, such as in work or school contexts), and the others (i.e., how one behaves within the close relationships with family and friends).

Here we created a questionnaire based on PREPOS’s model to investigate the presence of these seven personality categories in blind and sighted individuals in relation to COVID-19 pandemic. We explored their feelings and opinions about some life dimensions. Specifically, for each dimension listed below, participants were presented with seven statements and had to identify which reports (even more than one) better described themselves and their behaviour during COVID-19. There were seven possible statements per dimension, each of which was associated with one personality category. The questionnaire is reported in Table 3. The dimensions investigated were:

- Decision-making: the relationship between the internal decision-maker of the person and the external political decision-maker
- Rights: personal feeling about the interpretation of the central values (responsibility, justice, freedom, generosity, peace, humility, fidelity) according to a deontological ethic of duty
- Fun/pleasure: how the person shapes free time
- Negative experience with oneself: the most significant difficulties related to the relationship with oneself
- Positive/not negative experience with oneself: the positivity, the new awareness, the experiences of normality, referred to the relationship with oneself
- Negative experiences towards others: negative personal feelings in relationships with imagined or real others
- Physical activity: choices regarding physical activity
- Significant relationships: how the person experiences meaningful connections with others
- Future perspectives: the person’s expectations of what will happen to his or her life after COVID-19
- Social media: the personal exposure to social networks
- Time: the person’s perception of the passage of time

**Table 3:** Questionnaire 2 items: For each dimension (see rows), the researcher reads the seven statements, and the participants choose the ones better describing themselves during the lockdown.

	Stingy	Ruminant	Delusional	Partier	Apathetic	Invisible	Adhesive
Decision-making	If I could decide during this emergency, I would have made more rigorous decisions.	If I could decide during this emergency, I would have acted in advance, without stalling.	If I could decide during this emergency, I would have made more open decisions.	If I could decide during this emergency, I would offer at least free internet to everyone.	If I could decide during this emergency, I would have made the same decisions. In my opinion, gradually closing gave us time to understand and get used to this new situation.	I would have never put myself in the shoes of those who had to decide what to do during this emergency.	If I could decide during this emergency, I would have worried more about the life of families and children in this period of crisis.
Rights	In this emergency, we all need to be more responsible and do the things we are told and not those we would like.	In this emergency, everyone’s work is at risk. I do not accept that someone can work while others cannot.	Freedom is an irrepressible human right that today is compromised. I do understand the reason, but I disagree.	In this emergency, we are called to conscientiously donate what we have because donating is the primary means to support those who are really in need of our help.	This is finally the moment to experience peace and detach ourselves from daily stresses.	The ‘biggest crisis’ is the one each person is living with. Everyone’s experience must be listened to.	At this moment, the nation and its institutions should more concretely help all those that are helping others with their daily commitment.
Fun/pleasure	I use this period to exercise at home and take care of my diet.	Free time? What free time?	I enjoy expressing myself. Singing, drawing, writing, or creating something are all excellent means of doing so.	My aim in this period is constantly finding new ways of spending time.	Lying on the sofa, I listen to audiobooks, watch tv series, play online all day long.	Finally, I have the time for reading all the books I bought during the years, and I have never found time for reading.	I spend my free time calling or video calling my closest friends.
Negative experience with oneself	I am afraid of getting sick, and I will not take a single step out unless necessary.	It drives me incredibly crazy that I cannot do what I have planned and I still have in mind.	I cannot escape from this pain because everyone is similarly suffering.	I cannot find a point of reference to hold on to. It is a moment of confusion without exit. When I think I have finally grasped something, another doubt arrives, and I feel anxiety.	I feel I miss the peace I had before, and I must justify to others the reasons for my behaviour.	I feel I do not have any right to express my pain because of the more severe pain of others in this critical moment.	For me, living without hugs is like living without air.
Positive/not negative experience with oneself	Although I faced some difficulties too, I reorganized my daily routine straight away.	I am very busy, more than before.	I have a thousand ideas in mind. It is a very creative period.	With all the things I would like to do, I have no idea where to start. I switch from one thing to another constantly.	I feel good at home.	In this period, I empathise more often with others’ pain when I see some images or read the news.	I am happy when I manage to find a new way to feel closer to those I love.
Negative experience with others	I feel I am not protected enough when I go out, even though I carefully observe the preventive measures.	I often realize that others do not follow the restrictive measures as I do with a lot of effort. I hope that whoever is in charge gives them a fine.	In this period, people block the flow of my thoughts and drive me to focus on other situations I do not care about. People think about surviving in the present instead of dealing with what will come tomorrow, which is what matters.	I miss going out with friends, the jokes, the laughs. Alone, I feel lost and listless.	I am constantly involved meeting different requests, which gives me a lot of stress.	I avoid video calls because I am ashamed of others realizing how much I am suffering.	In these difficult moments, it is possible to evaluate who are those that really matter and are honest in their friendship.
Physical activity	Every day I exercise at home. I keep stress under control.	In this period in which many live inactively, I planned during the week specific daily moments for sport: jogging, aerobic activity, bodyweight training, weightlifting, and much more.	I should exercise more, but then I get lost in things that happen, and what I feel like doing during the day…and suddenly it is the end of the day.	I run at home, I behave as if I have gym equipment, and I do all this in video calls with friends.	Finally, I am not forced to exercise, and I can do what I like most or nothing at all.	I stopped exercising as I was doing before. I look for other ways to move my body more in line with my feeling.	I do not care what kind of physical activity I do, but it is essential to plan it together with friends or partners, in video calls, and have a snack together afterwards.
Significant relationships	It is essential to manage our critical relationships responsibly. In this challenging period, we are asked to make sacrifices.	I always manage to engage in significant and flourishing reconnections.	In my significant relationships, I often offer my ideas and inspirations. My thoughts are always very active.	In my significant relationships, I showcase a joyful and hopeful emotional state.	In my significant relationships, I keep calm, and I am patient.	I am always listening to myself, and I listen carefully to others too.	I am very grateful to have the significant relationships I chose, or that life offered me right now. I appreciate them even more.
Future perspectives	After COVID-19, I will return to my old activities, even though nothing will be as before.	What we will do after this period depends on what we are doing now.	After COVID-19, I believe that everything will be different. Our approach to life will be completely transformed.	I have no ideas about how life after COVID-19 is going to be, but I feel it will be very exciting.	What is going to happen after COVID-19 is not something we should worry about now.	After this period of COVID-19, I think my life will be the same as my life now.	After this period of COVID-19, hugs, kisses, cuddles will have other meanings, and I am looking forward to experiencing them with those I love.
Social media	I post only photos from the past on social media.	I post only useful information on social media.	I post interesting links on social media.	I post jokes on social media.	I post photos where only details or specific parts of a whole are represented.	I avoid posting anything on social media, but I prefer posting images of nature if I must.	I post receipts, food, or brands.
Time	I plan my daily time based on the things I must do. I give myself specific goals, and I organize my time consequently.	There is never enough time to do everything I want to during the day. I feel like I am always in a race against the clock. Usually, at the end of the day, there is something I wanted to do that I have not had time to do yet.	The passage of time changes based on my thoughts: sometimes time passes very slow, sometimes it passes very fast.	Living in the present for what it gives us, the highest and the lowest moments, is all that matters: the past has gone, and the future has yet to arrive.	I feel I have all the time I need. I know boredom very well, and this does not scare me at all.	The rhythm of everyday life rarely adapts to my inner rhythm.	Time passes quickly when I am with the ones I love; it never passes when I interact with those I dislike.

Each statement refers to one specific personality category (see columns).

### Statistical analysis

For the open-ended question, the scientists who scored the transcripts did not know whether a response sheet was from a blind or sighted person. To analyse responses, we first defined *a priori* four subcategories of interest: social aspects, logistical aspects, aspects related to new norms, and psychological aspects. Then, three different researchers read the transcripts of each participant and attributed the reported difficulties to one of the four subcategories. For each mentioned difficulty attributed to a specific category, one point was assigned to that subcategory. Two researchers out of three needed to agree, otherwise we would have created another subcategory named “Others.” Subsequently, within each subcategory we identified specific dimensions to sum up the responses without being too vague and giving a more specific idea about the type of difficulty faced within the general subcategory. Thus, we referred to the following subcategories and dimensions: (1) Social aspects: social impairment, forsaking having fun, not going out; (2) Logistical aspects: functional reorganization, difficulty with work activities, loss of autonomy; (3) Aspect of new norms: difficulty with self-protection, difficulty with social distancing, difficulty with transportation; (4) Psychological aspects: negative psychological experiences. Responses were qualitatively compared between groups. For the open-ended question, no statistics were applied.

For Questionnaire 1, results of questions with Likert scale or actual estimation numbers were analysed by comparing groups through nonparametric t-tests. Since we were interested in changes associated with COVID-19, when the same questions addressed both the current moment and life before COVID-19, the differential scores were calculated as subtractions (i.e., scores at the present moment minus score before COVID-19). For questions on Questionnaire 1 requiring Yes/No answers, the percentage of individuals reporting “Yes” was calculated for each group and qualitatively compared.

For Questionnaire 2, scores were obtained by summing up the number of times participants selected statements belonging to a specific personality category for each group. For each personality category, comparisons were made between groups through non-parametric t-tests.

## Results

### Preliminary open-ended question

**Figure 1:**
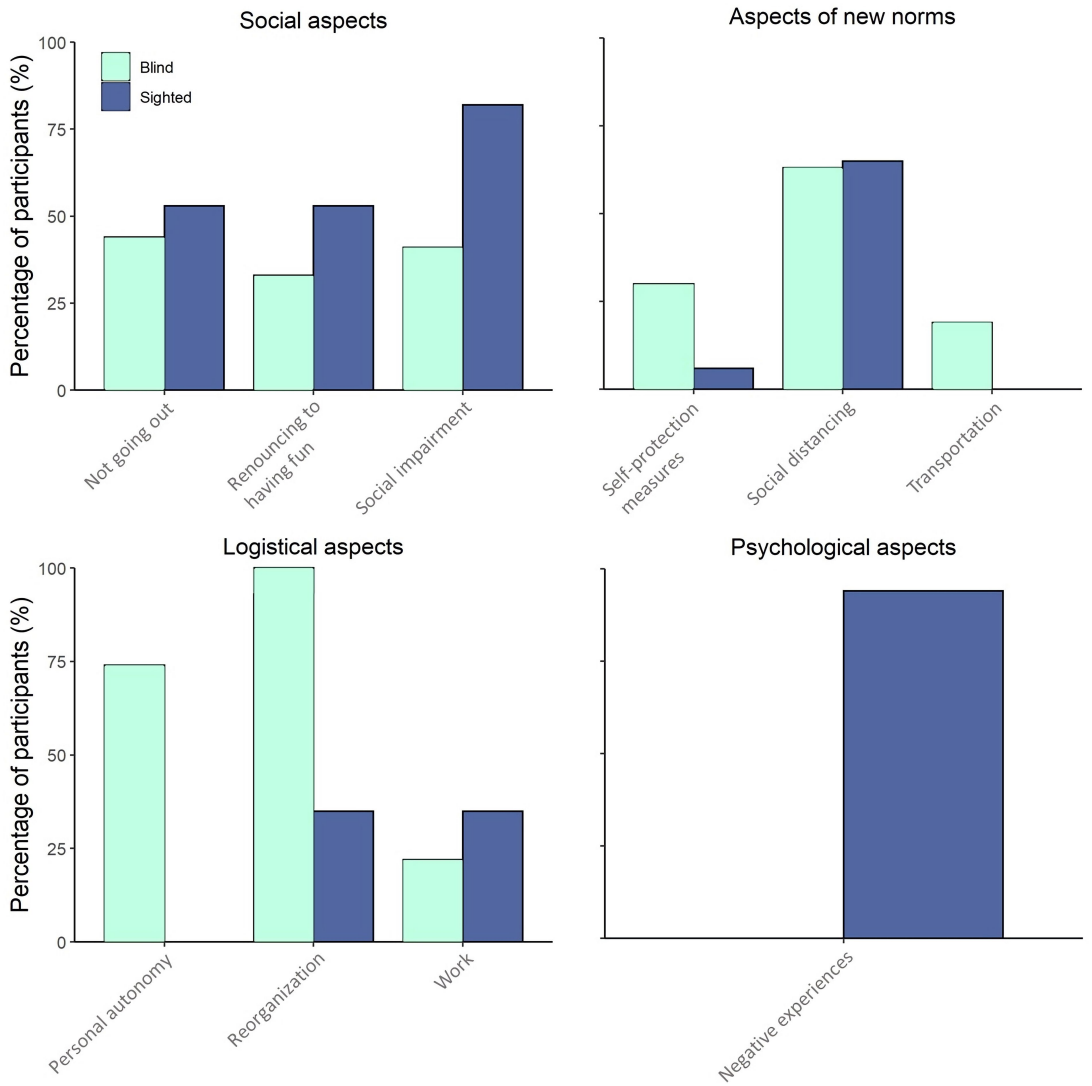
Results of the open-ended question: percentage of participants answering the open-ended question and reporting difficulties in the following categories: social aspects, aspects of new norms, logistical aspects, and psychological aspects. Each category is divided into different dimensions related to the main topic of that category. In light blue is the blind group; in dark blue is the sighted control group.

Qualitatively, we noticed that blind individuals provided longer and more detailed responses to the open-ended question, while sighted persons produced faster and shorter answers. The results showed differences between the sighted and the blind groups in their experience with COVID-19 (Figure 1). In all dimensions of the ‘social aspects’ subcategory, sighted individuals were more affected by COVID-19 restrictions than blind participants. More specifically, sighted individuals reported more difficulties related to social impairment, i.e., the limited possibility of seeing family members and friends (82% of sighted participants versus 40% of blind participants). They also complained more about giving up on fun activities (52% of sighted participants versus 33% of blind participants) and about not going out (52% of sighted participants versus 44% of blind participants). Blind participants, on the other hand, reported more problems than sighted individuals in the ‘logistical aspects’ subcategory. All blind participants stated they had difficulties with the reorganization of daily activities, while less than half of sighted participants indicated the same impediments (100% of blind participants versus 35% of sighted participants). In blind participants, difficulties in reorganization mainly concerned shopping, routine activities, cleaning the house, and giving up on sports activities. Curiously, only blind participants (74%) reported a loss of personal autonomy, which was not mentioned by any of the sighted participants. This observation suggests that in the absence of vision, personal independence became a particularly significant aspect of a person’s life during COVID-19. However, a certain level of discomfort was also caused by difficulties related to work, and this was slightly more perceived by sighted people (35% of sighted participants versus 22% of blind participants). Mixed results emerged from the participants’ responses regarding the norms imposed by the government to prevent the spread of the virus. Both groups similarly experienced significant distress associated with social distancing (64% of sighted participants versus 62% of blind participants). However, blind participants declared higher discomfort in relation to self-protection than the sighted control group (6% of sighted participants versus 29% of blind participants), and the use of public transport was an aspect only mentioned by the blind group (18%). Finally, in the ‘psychological factors’ subcategory, we found that only sighted participants reported a very high percentage (94%) of negative psychological experiences, such as feelings of loneliness, anxiety, and fear about the isolation they were experiencing.

### Questionnaire 1

**Figure 2:**
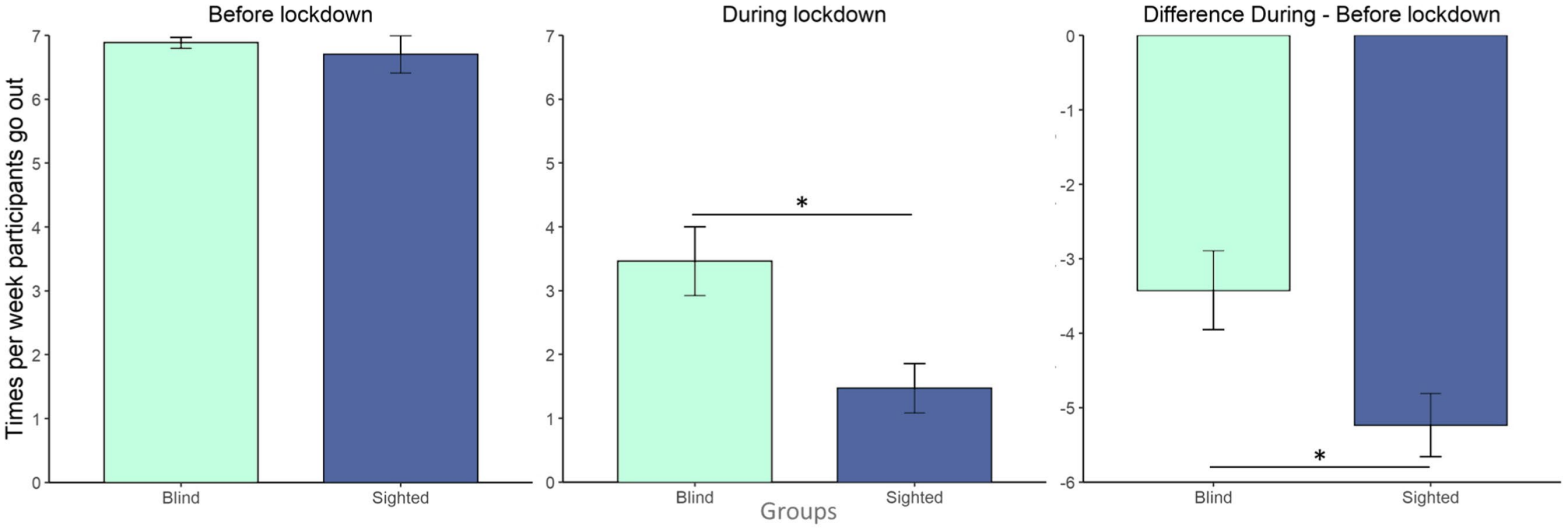
Number of times per week participants go out: bar plots show the number of times per week participants went out from home divided per group (in light blue blind group; in dark blue sighted controls group) before the lockdown (left panel), during the lockdown (central panel) and the difference between during and before the lockdown (right panel, negative values mean fewer outings during the lockdown). Error bars show the standard error of the mean. *: value of *p* <0.05.

Both blind and sighted participants declared that their daily routine was in general modified due to COVID-19 (average score: 6.5/10 for blind individuals, 7.1/10 for sighted individuals), but no differences between the groups were found (Mann–Whitney test: W = 195, value of *p* = 0.52). However, while sighted and blind individuals reported a similar number of outings from home before the COVID-19 (W = 218, value of *p* = 0.888), sighted people, compared to blind participants, went out significantly less during the lockdown (W = 313.5, value of *p* = 0.019; Figure 2). The difference in times participants were going out from home before and during the lockdown was always negative, suggesting that the frequency of going out decreased with the pandemic for both groups. However, this difference varied between the two groups as it was greater for sighted participants, who significantly reduced their outings during COVID-19 (W = 134, value of *p* = 0.028). As for social content, both groups similarly reported keeping in touch virtually with the same amount of people (W = 268, value of *p* = 0.214), but physically meeting drastically fewer persons (W = 236.5, value of *p* = 0.561).

Sleep habits were also affected by COVID-19. Indeed, both groups reported that their sleep quantity and quality slightly changed during the lockdown (average score for quantity: 2.4/10 for blind individuals, 4.1/10 for sighted individuals; the average score for quality: 3.3/10 for blind individuals, 4.8/10 for sighted individuals). Yet although the perceived quality of sleep was similarly affected (W = 157.5, value of *p* = 0.112), a difference between blind and sighted individuals appeared for perceived change in the quantity of sleep (W = 127.5, value of *p* = 0.018). Sighted people felt a more significant modification in terms of hours of sleep during COVID-19 compared to before the lockdown and declared encountering difficulties in falling asleep and waking up during the night more often than blind participants. Specifically, the percentage of people who found it more challenging to fall asleep during COVID-19 pandemic increased by 19 and 52% for blind and sighted participants, respectively (percentage of people who needed a lot of time to fall asleep before COVID-19: 34.6% for blind individuals, 11.8% for sighted individuals; percentage of people who needed a lot of time to fall asleep during COVID-19: 53.8% for blind individuals, 64.7% for sighted individuals). Similarly, the percentage of people who reported waking up more often at night during lockdown increased by 11 and 35% for blind and sighted participants, respectively (percentage of people who often woke up at night before COVID-19: 34.6% for blind individuals, 11.8% for sighted individuals; percentage of people who often woke up at night during COVID-19: 46.2% for blind individuals, 47% for sighted individuals). Overall, these percentages seem to increase more for sighted than for blind people. In contrast, only 6% of sighted participants reported waking up earlier during the lockdown, against 15% of blind participants (percentage of people who woke up earlier before COVID-19: 30.8% for blind individuals, 29.4% for sighted individuals; percentage of people who woke up earlier during COVID-19: 46.2% for blind individuals, 35.3% for sighted individuals). Moreover, when sighted individuals were asked to evaluate the number of hours of sleep per night (before lockdown: mean ± SD: 7.02 ± 1.25; current moment: mean ± SD: 7.38 ± 1.29), there was no difference compared with blind participants (before lockdown: mean ± SD: 6.51 ± 1.32; current moment: mean ± SD: 6.48 ± 1.62) for both the current moment (W = 163, value of *p* = 0.146) and the period before the pandemic started (W = 168.5, value of *p* = 0.186). In line with this, the differential scores in hours slept per night at the current moment and before COVID-19 were similar between groups (W = 238.5, value of *p* = 0.644).

### Questionnaire 2

**Figure 3:**
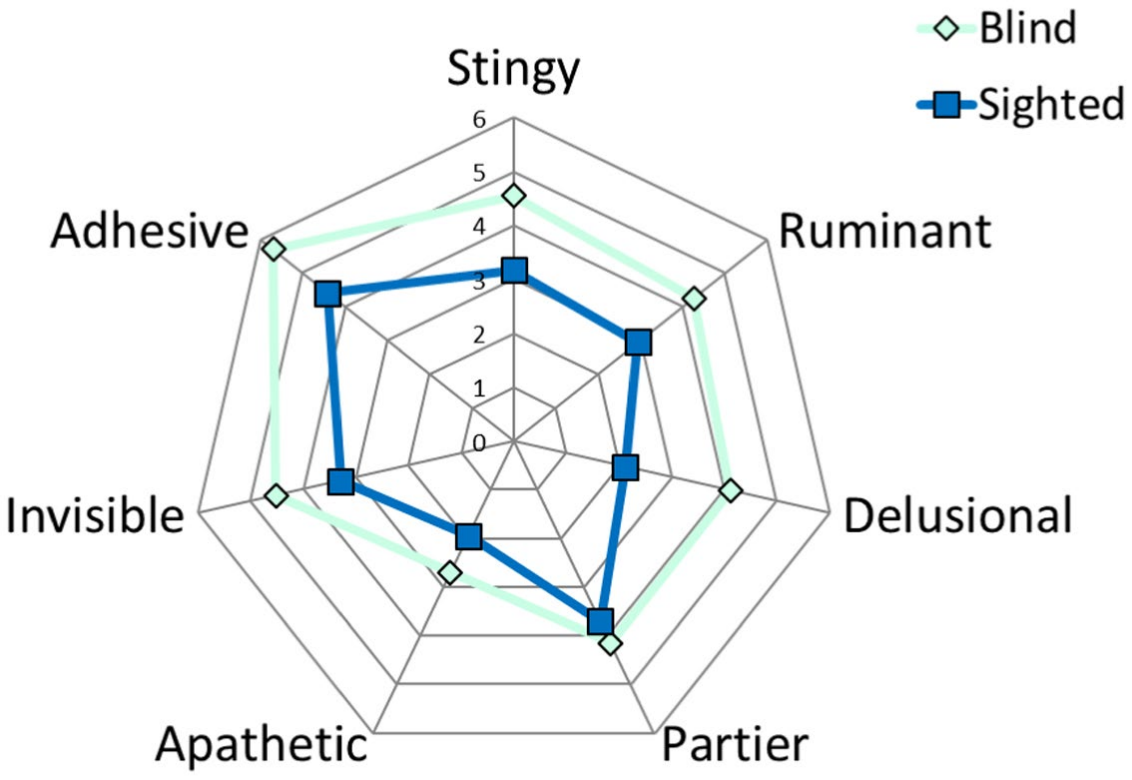
Graphical representation of the average number of responses to questionnaire 2 for each personality category. The dark blue line represents sighted participants; the light blue line represents blind participants.

According to Questionnaire 2, there were some differences in personal experience and relationships between sighted and blind individuals during COVID-19. Table 4 reports an average number of responses for blind and sighted participants for each personality category. Specifically, we calculated for each group and averaged the number of times participants selected statements belonging to a specific personality category. As highlighted by the last column, blind participants tended to choose overall more items (average number of selected statements: 30 for blind participants, 21.59 for sighted participants). These results are also displayed in Figure 3. The broader area for blind people, which contains the perimeter of sighted people, indicates more responses for this group. It is also interesting to note the similar trend between the shapes of blind and sighted individuals in Figure 3, with the higher scores for both groups for the ‘adhesive’ personality and the lower score for both groups for the ‘apathetic’ personality. Instead, the category for which the shape most differs between groups is the ‘delusional’ personality. For the ‘partier’ personality, sighted individuals show peak and present scores that are more like blind individuals.

**Table 4:** Average number of responses to questionnaire 2.

	Stingy	Ruminant	Delusional	Partier	Apathetic	Invisible	Adhesive	Total
Blind	4.555556	4.59259	4.111111	4.148148	2.703704	4.518519	5.703704	30
Sighted	3.176471	2.941176	2.117647	3.705882	1.941176	3.294118	4.411765	21.58824

For each personality category, the mean number of times participants selected statements belonging to a specific personality category is shown for blind and sighted groups. The last column represents the mean number of statements chosen considering all types together.

In line with Table 4 and Figure 3, blind people statistically showed significantly higher scores than sighted people for the ‘stingy’ (W = 122, value of *p* = 0.004) and ‘delusional’ (W = 93.5, value of *p* = 0.0009) personalities. Although slightly less than for ‘stingy’ and ‘delusional’ personalities, scores were higher for blind participants for ‘ruminant’ (W = 141.5, value of *p* = 0.03), ‘invisible’ (W = 145, value of *p* = 0.04), and ‘adhesive’ (W = 139.5, value of p = 0.03) personalities. Similar results were obtained between groups for the ‘partier’ (W = 198, value of *p* = 0.4) and ‘apathetic’ (W = 162, value of *p* = 0.09) personalities.

## Discussion

### Results of the preliminary open-ended question

The answers to the open-ended question suggest that for the sighted participants, social aspects comprised most of the problems associated with the restrictions and isolation imposed by the COVID-19 emergency. Instead, problems for blind individuals were mostly practical. This is particularly reflected in the asymmetrical responses. All sighted people reported a negative psychological experience probably associated with social distancing, while no blind people did. The finding that psychological and social issues were most impactful on sighted people is in line with previous research reporting a significant increase in level of stress, depression and anxiety symptoms in people with no disabilities during the COVID-19 pandemic (Benke et al., 2020; Ozamiz-Etxebarria et al., 2020; Pierce et al., 2020; Evans et al., 2021). In our study, sighted individuals were disappointed by the limited possibilities of seeing family members and friends and giving up on fun activities and going out. For example, sighted individuals declared that the most significant difficulties they encountered in their everyday life during the lockdown were *“not being able to physically see other people,” “the insecurity and fear of not being able to hold up,”* and also “*the need for normality*.”

On the contrary, the blind respondents reported a loss of personal autonomy, which fits with the overall emergent theme of logistical issues proving most challenging for this group. Blind participants complained, for example, of *“the need of always going out accompanied,” “the obligatory routes in the shops and public places that, if I cannot see, I do not know where I have to go,”* and *“the difficulty of shopping online because it takes a long time to place the order.”* The reports are in line with those acquired by the survey of the Royal National Institute of Blind People,^2^ in which blind and partially sighted respondents declared they feel less independent than before the lockdown and that this aspect was mainly related to the need for a guide to go out. This lack of independence might be exacerbated by the fact that 25% of the interviewees declared that they could not count on a person who could guide them daily, even while 49% of the blind respondents had someone shop for them, an increase from 18% pre-pandemic. Another logistical issue emerged in public transport, which *“becomes impossible to take without a companion, and this bothers me a lot because it terribly limits my autonomy which is so difficult to achieve.”* This finding reflects that of a recent study revealing that people with visual impairment in Colombia reported barriers to mobility when taking public transportation. Specifically, they had trouble with not touching surfaces on the transports, not finding pedestrians willing to help, and not being able to keep a physical distance (Oviedo-Cáceres et al., 2021). Even gloves have been reported as barrier between the skin and external stimuli that generated practical distress for blind individuals as interfering with tactile perception (Rizzo et al., 2021).

All these aspects highlight the enormous change caused by the pandemic for people with visual impairments who already had to deal with many practical barriers. The requirement to maintain social distancing in the daily life of a person with visual impairment translates to a loss of support, both in an affective sense and in a more purely practical one, as when, for example, other people no longer intervene to help due to a need to maintain distance (Rizzo et al., 2021). The presence of strong negative feelings, such as loneliness, in sighted participants but not in blind people could be due to the fact that loneliness already characterizes life of visually impaired people even before COVID-19 (Verstraten et al., 2005; Alma et al., 2011; Brunes et al., 2019b) and therefore they could have felt less the impact of the pandemic on this aspect (Heinze et al., 2021b).

### Results of questionnaire 1

The daily routine of all participants involved in the study was drastically modified by COVID-19 restrictions. Blind and sighted people reported going out at similar rates before COVID-19, and both reduced the frequency of leaving the house during the pandemic. Other studies similarly reported increases of social isolation across groups during the pandemic (Smith and Lim, 2020; Murayama et al., 2021). This is in line with distress reported by both groups related to the impossibility of going out in the preliminary open-ended question; one sighted participant complained of the difficulty of *“staying at home not by choice, but by obligation,”* while another blind person noted it was difficult *“accepting not being able to leave the house if not accompanied.”* However, sighted people appeared to go out significantly less often during the lockdown than blind people. Considering the findings on the open-ended question, this difference is likely explained by the difficulties faced by blind people in reorganizing the practical aspects of their life at home. It is plausible that without going out, blind people could not take care of primary care needs, such as getting groceries. Accordingly, the Royal National Institute of Blind People of UK asserted that their helpline support service received an average of over 100 calls a day on food-related problems experienced by blind people who could not, for example, use the online websites for shopping or visit their typical shops as they were closed due to restrictions.^3^ Going out less is also linked with fewer in-person social interactions among both groups, which in turn likely triggered the discomfort associated with social impairment (such as ‘having fun’ among sighted people) as seen in the results of the open-ended question.

The COVID-19 pandemic also seemed to affect sleep quantity and quality in both groups. This is in line with the literature, which suggests that self-reported sleep patterns, sleep duration, and sleep quality have all worsened during periods of lockdown (Gupta et al., 2020; Perez-Carbonell et al., 2020) and that there are no statistically significant differences in sleep quality between people with and without visual disability during the pandemic (Heinze et al., 2021a). Although sleep issues characterize the life of blind people prior to COVID-19 (Tamura et al., 2016; Peltzer and Phaswana-Mafuya, 2017), the pandemic appeared to not have a significant greater impact on them. However, in our study, sighted participants reported a more significant change in their sleep quantity compared to blind individuals, with the former group declaring more issues in falling asleep and maintaining regular sleep throughout the night. This finding accords with a recent study that reports significant deterioration in sleep quality in participants without visual disabilities compared with those with visual impairment (Peltzer and Phaswana-Mafuya, 2017). Although there is a difference in perception, we do not find actual differences in hours slept per night were reported between groups. This apparent contradiction could be interpreted considering the negative psychological experience reported by most sighted participants in the open-ended question, such as a *“sense of loneliness,”* and *“fear for the future.”* Even though no effective differences in sleep hours emerged, it could be that when asked to think about their sleep habits, sighted people have been influenced by the negative feelings and psychological fatigue experienced during the lockdown. It is known that anxiety, for instance, impacts on sleep quality (Altena et al., 2020; Xiao et al., 2020; Evans et al., 2021).

To sum up, Questionnaire 1 suggested that the COVID-19 emergency impacted the daily routine, social life, and sleep habits of sighted and blind people. It highlighted in particular that the number of times blind people went out was not significantly reduced during the lockdown, no matter what the governmental restrictions were. This is interesting and makes us hypothesize that blind people were forced to go out to overcome some practical problems they could not solve at home due to their visual impairment. At the same time, sighted individuals reported more negative experiences related to sleep habits.

### Results of questionnaire 2

The results from Questionnaire 2 suggest that blind and sighted individuals involved in the study experienced some differences in their relationships with themselves and others during COVID-19. Blind people statistically were more represented than sighted people among the ‘stingy’, ‘delusional’, ‘ruminant’, ‘invisible’, and ‘adhesive’ personality categories. Both blind and sighted groups were represented equally among the ‘apathetic’ and ‘partier’ categories. Since the PREPOS model received attention mainly in Italy and this is a home-built questionnaire, it is important to state that the findings of Questionnaire 2 are qualitative, and observations have a speculative nature.

The ‘stingy’ personality category represents control and fear as the primary emotion. This category’s keywords are organization, rigor, tidiness, responsibility, protection, and attention. In a recent interview, a blind researcher highlighted the discomfort caused by the restriction to touch others, as senses such as touch are the primary means for monitoring aspects of one-self and the external world for blind people (Giudice, 2020). The higher score of blind people for the ‘stingy’ category could imply that the restrictions due to COVID-19, such as the one about touch, induced in blind people a more vital need for monitoring and keeping control of their lives and their social relationships. This is in line with the findings of the open-ended question, which showed that blind individuals had to concentrate more and put more effort into the reorganization of their life, with evident distress associated with difficulties in self-protection.

The other personality category that differs between groups is ‘delusional’, based on mentalization and detachment. The keywords of this category are openness, freedom, escape, ideas, creativity, thoughts, breaks, and diversity. The higher score for blind people may suggest that blind people focused more than sighted people on their mental and emotional lives and distanced themselves more from reality. This fits with the loss of personal autonomy suggested by responses to the open-ended question. Contrarily, the lower score for sighted people may be linked to the negative personal experiences they reported in the open-ended question, which are in line with studies reporting a significant increase in levels of self-reported depression symptoms in people with no disabilities (e.g., Casten et al., 2010; Evans et al., 2021). Sighted individuals may have fostered less mental activity, stuck with the external restrictions, and have been involved in more practical activities that were difficult to access for blind people, such as online physical exercises (Richardson et al., 2022).

In addition, the ‘ruminant’ category was represented more highly among blind participants. It is a personality based on action and has anger as its main emotion. The critical words for ‘ruminant’ are anticipation, work, getting angry, sacrifice, effort, growing, using, and doing. We hypothesize that restrictions due to COVID-19 provoked a more robust reaction of anger in blind people. It could be that blind people experienced more unfairness in how society took care of their condition during the emergency. In Rizzo et al. (Rizzo et al., 2021), the authors emphasized the lack of a viable solution to overcome the repercussions of social distancing and minimization of touch for blind people. This could have led to a stronger sensation of frustration and deprivation that caused greater representation of the ‘ruminant’ personality category.

Similarly, another personality category showing a mildly higher score for blind individuals was ‘invisible’, a personality based on sensitivity and embarrassment to express oneself at an emotional level. The keywords better describing this category are crisis, pain, listening, reading, empathizing, agony, feeling, avoiding, and inner life. As with the ‘delusional’ personality category, this result suggests that blind people could have focused more on themselves during the period of lockdown, paying more attention to their feelings and emotions.

The last personality that shows a slightly higher score in blind people is ‘adhesive’, which represents a personality based on interaction with others, and which is characterized by attachment as the core experienced emotion. The keywords are relationships, gratitude, hugs, kisses, cuddles, food, and love. The higher scores of blind people may mean that during the restrictions due to COVID-19, blind people experienced a more vital need to interact with significant others. In line with the logistical difficulties highlighted by responses to the open-ended question, we can speculate that this personality category is more represented among in blind people because they needed more practical help and lacked physical contact during social interactions, which, in turn, interfered with social inter-personal communication and emotional expression (Giudice, 2020).

The two categories showing similar results between the groups were ‘apathetic’ and ‘partier’. ‘Apathetic’ describes a personality based on detachment and tranquillity at an emotional level. The keywords of this personality are annulment, gradualness, calmness, justification, nothingness, patience, and boredom. The similar representation of this personality among the two groups may underlie a similar experience of detachment and calm during the lockdown. ‘Partier’, on the other hand, is a personality based on involvement and pleasure. The keywords are free, donate, entertain, agony, confusion, emptiness, joy, emotional, jokes, present. Similar representation among blind and sighted groups suggest that all participants lived this pandemic period with an equivalent level of involvement.

Despite each specific category, blind people report overall a higher number of items selected. This may mean that blind participants were more involved in the questionnaire; they appreciated being contacted and participating in the study. In line with their scores in the ‘invisible’ personality, they may have enjoyed exploring their personal experiences, emotions, and feelings during this period, where they also could have felt neglected by the system. By observing the overall shapes of the graphs reporting the results (Figure 3), we can add that the pattern is similar between the groups, indicating a comparable general reaction between blind and sighted people in their responses to COVID-19.

## Conclusion

The present study reveals that the COVID-19 pandemic caused problems and challenges in people both with and without visual impairment. All participants were forced to change their daily habits, such as by going out less, having fewer in-person social interactions, and experiencing alterations in sleep routine. However, the lockdown impacted sighted and blind individuals’ lives differently.

For sighted people, their personal and social spheres were mostly affected. The open-ended question results suggested negative psychological experiences and distress associated with the social dimension, while Questionnaire 1 indicated perceived difficulties in sleep habits and responses to Questionnaire 2 lower scores for interaction and attachment. For blind individuals, struggles were more related to the practical side, as demonstrated in i) the open-ended question that highlighted issues associated with reorganization, loss of personal autonomy, self-protection measures, and use of public transport, ii) Questionnaire 1, which demonstrated that blind persons needed to go out more often than the sighted, and iii) Questionnaire 2, where blind respondents had higher scores for interaction. If, on the one hand, blind people seem to have more practical issues with the external world, at an emotional level, they were more in touch with their inner world, which was demonstrated in their higher Questionnaire 2 scores for mentalization and sensitivity. Blind participants seemed to experience more need of control and higher levels of fear, likely associated with the practical daily challenges they had to face and anger, likely associated with the lack of consideration from the system. Both these conclusions are supported in the results of Questionnaire 2.

Some limitations need to be considered when interpreting these results. Sampling is the first issue. The small sample size, lack of information about level of education and living situation of respondents, and the fact that genders were unequally represented between sample groups all limit the strength of these findings. Indeed, although no differences between females and males have been observed in previous studies investigating the effects of COVID-19 in blind people (Gombas and Csakvari, 2021; Suraweera et al., 2021; Richardson et al., 2022), this does not permit us to rule out the possibility that group differences between blind and sighted were driven by a gender effect. Other issues that limit the implications and generalization of this research are related to the use of questionnaires generated for the purpose of this study and the use of studies and models by the PREPOS group. The work by PREPOS is relatively new, few and only Italian studies exist evaluating it and, consequently, our findings related to Questionnaire 2 need to be interpreted qualitatively. Indeed these findings are mostly speculations and further research is needed (Masini, 2005a,b).

To conclude, COVID-19 changed everyone’s lives, but this has been experienced differently between people with and without visual impairments. Our findings should be considered when developing intervention programs to support and improve the quality of life of visually impaired people, especially as this period of emergency has not yet passed. Logistically, concrete actions should be planned to take care of blind people in everyday needs. For instance, social assistance could be provided for supporting blind people when they need to go out, with an emergency number that can be used to solve practical issues such as troubles with public transportation or food delivery. From a technological point of view, Rizzo and Giudice (Rizzo et al., 2021) reviewed the assistive devices that could help blind people move around safely even during COVID-19 pandemic. The VISION platform (Visually Impaired Smart Service System for Spatial Intelligence and Onboard Navigation) could be optimized to support real-time situational and obstacle awareness for socially distanced travel (Amedi et al., 2005). While these are only a few practical measures that could be implemented, our results suggest that societies globally should strongly consider the needs of people with sensory impairments when dealing with crises and changes to norms that unavoidably impact everyone.

## Data availability statement

The dataset presented in this study can be found in online Zenodo repository at the following link: https://doi.org/10.5281/zenodo.7144037.

## Ethics statement

The studies involving human participants were reviewed and approved by Comitato Etico, ASL3 Genovese, Italy. The patients/participants provided their written informed consent to participate in this study.

## Author contributions

MG, GB, EM, and MA contributed to conception and design of the study and performed the statistical analysis. EF collected the data and organized the database. MG, GB, EM, EF, and MA wrote the manuscript. All authors contributed to the article and approved the submitted version.

## Funding

The research is partially supported by the MYSpace project (PI MG), which has received funding from the European Research Council (ERC) under the European Union’s Horizon 2020 research and innovation program (grant agreement no. 948349).

## Conflict of interest

Author EM was employed by company PREPOS Studio Associato, Lucca, Italy.

The remaining authors declare that the research was conducted in the absence of any commercial or financial relationships that could be construed as a potential conflict of interest.

## Publisher’s note

All claims expressed in this article are solely those of the authors and do not necessarily represent those of their affiliated organizations, or those of the publisher, the editors and the reviewers. Any product that may be evaluated in this article, or claim that may be made by its manufacturer, is not guaranteed or endorsed by the publisher.

## Footnotes

^1^ https://www.rnib.org.uk/campaigning/priority-campaigns/inclusive-journeys/effect-lockdown-and-social-distancing-blind-and-partially-sighted-people/#:~:text=Two%2Dthirds (66%25) of,as them who can guide

^2^ https://www.rnib.org.uk/campaigning/priority-campaigns/inclusive-journeys/effect-lockdown-and-social-distancing-blind-and-partially-sighted-people/#:~:text=Two%2Dthirds (66%25) of,as them who can guide

^3^ https://www.rnib.org.uk/campaigning/priority-campaigns/inclusive-journeys/effect-lockdown-and-social-distancing-blind-and-partially-sighted-people
